# Impact of Alzheimer’s Dementia on Caregivers and Quality Improvement through Art and Music Therapy

**DOI:** 10.3390/healthcare9060698

**Published:** 2021-06-09

**Authors:** Laura-Cristina Popa, Mihnea Costin Manea, Diana Velcea, Ion Șalapa, Mirela Manea, Adela Magdalena Ciobanu

**Affiliations:** 1 Department of Psychiatry, ‘Prof. Dr. Alexandru Obregia’ Clinical Hospital of Psychiatry, 041914 Bucharest, Romania; pcristina9025@gmail.com (L.-C.P.); diana.velcea@gmail.com (D.V.); ionsalapa@gmail.com (I.Ș.); mirelamanea2003@yahoo.com (M.M.); 2 Department of Psychiatry and Psychology, ‘Carol Davila’ University of Medicine and Pharmacy, 020021 Bucharest, Romania; 3 Neuroscience Department, Discipline of Psychiatry, ‘Carol Davila’ University of Medicine and Pharmacy, 020021 Bucharest, Romania

**Keywords:** dementia, Alzheimer’s, music therapy, art therapy, caregivers, quality of life, non-pharmacological, alternative therapy, clinical trials

## Abstract

Dementia is a general term for a series of medical conditions that affect the brain and evolve progressively. According to the literature, there are over 200 subtypes and causes of dementia, with Alzheimer’s disease (AD) being the most common in elderly people. AD is an irreversible progressive neurodegenerative condition that leads to a decline in mental function, enough to disrupt daily life. Thinking skills slowly deteriorate, which, in advanced stages, makes it impossible to perform simple tasks. Besides the change in the quality of life of AD patients and their families, there is a considerable alteration in the quality of life of their caregivers, whose health can be negatively affected by the development of mental and somatic disorders. This article reviews the literature in order to reveal the benefits of applying non-pharmacological interventions such as music and art therapy to improve quality of life. This article also aims to shed light on the impact of this disease on the caregiver’s life. Music and art therapy have produced reliable results in the treatment of patients with AD, and the best effects are related to increased socialization and the maintenance of social status.

## 1. Introduction

Dementia, a condition studied for many years, is defined as a group of diseases characterized by the global, chronic, progressive, and irreversible intellectual deterioration of the entire psyche, mainly affecting cognitive functions, emotional life, and social behavior. According to the literature, Alzheimer’s disease is the leading cause of dementia. Data on the neurodegeneration of the cholinergic system and molecular pathogenic aspects were obtained following the discovery of amyloid β (Aβ) and Tau proteins, but these are not sufficient to elucidate the cause [1,2].

Dementia in Alzheimer’s disease is a significant concern in medical and social fields, impacting the people diagnosed, their relatives, and society. People diagnosed with this disorder experience progressive cognitive decline, a functional deficit that alters life quality. Thus, a complex care plan is required, which entails financial and emotional burdens. Cognitive and non-cognitive symptoms associated with emotional effects may cause psychosomatic disorders among relatives [3].

Numerous publications in the literature have provided data on dementia, and research has often focused on developing therapeutic guidelines for treating the symptoms associated with the disorder and dementia per se. Pharmacoeconomic research is also of particular importance in Alzheimer’s dementia, as it provides information on financial implications and therapeutic aspects. Recently, both therapeutic companies and mental health professionals have expressed a need for dementia assessment tools [1,4]. Assessment tools can provide data on the impact of Alzheimer’s disease on the “overall capacity” of the person diagnosed with the condition, its evolution, and the therapeutic response. Furthermore, assessments provide fine-grained data on the patient’s perspective of how the disease affects him or her [5].

Quality of life represents a “multidimensional concept” and is the subject of global discussion [1]. Over time, numerous quality-of-life assessment scales have been developed for this risk group, but these measurement tools require the administrator to perform them well.

People with Alzheimer’s dementia also suffer from behavioral disorders that reduce their quality of life and that of their families. The evaluation of these patients is complex: in contrast to patients with other psychiatric pathologies, patients with Alzheimer’s dementia often have limited ability to express themselves, and they can have difficulty navigating a complex system of mental and physical help.

In the Classification of Mental and Behavioral Disorders ICD-10, Diagnostic Manual and Statistical Classification of Mental Disorders (DSM-V), and the literature, Alzheimer’s dementia is categorized in the group of chronic neurodegenerative pathologies, which are characterized by an insidious onset and a slow progressive decline. The medical field recognizes dementia in Alzheimer’s disease as the most common type in this category [6].

The associated behavioral symptoms are characterized as intrinsic, and with the evolution of the disease, it becomes increasingly difficult to manage [7].

Caregivers face multiple issues associated with major psychological, physical, and financial burdens. Many studies have shown that caregivers experience alterations in their physical condition secondary to the action of chronic stress. They can develop cardiovascular disease, and especially, high blood pressure [8].

Despite efforts to treat this neurocognitive disorder, no curative therapies have been found, and with the development of dementia, the level of fragility of these patients increases [9]. Alleviating the suffering of these patients requires effective interdisciplinary collaboration and, importantly, an open relationship with family members for the most beneficial management.

The World Health Organization first introduced the concept of quality of life in 1947 and defined it not only as a lack of disease and infirmity but also as a state of well-being. According to data published by Post in 2014, the quality-of-life concept was introduced to the literature in 1960, and in 1975, it officially became a crucial term in the medical literature database [10].

According to Olazarán et al., non-pharmacological interventions in people with dementia positively impact their quality of life. However, the evaluation of this multi-factorial concept involves several challenges [11].

Assessment scales are applied largely by caregivers, which can lead to an underestimation of the patient’s quality of life, typically due to a hasty evaluation because of caregiver burnout and the labile mood of their patients [12].

## 2. Materials and Methods

This study aimed to review the literature from the period 2000–2021 on the steps taken to study quality of life in people affected by Alzheimer’s dementia after receiving non-pharmacological interventions. For this review, searches of the literature were performed in databases such as Google Scholar and PubMed. For this research, the narrative literature review style recommendations were followed [13]. Keywords such as “dementia”, “Alzheimer’s”, “music therapy”, “art therapy”, “quality of life”, “non-pharmacological”, “alternative therapy”, and “clinical trials” were used in the search engines. Articles written in English were reviewed.

During the research, the principal articles’ bibliographies were also checked to find relevant data.

In the process of selecting the relevant materials for this paper, we used the following inclusion criteria: articles written in the English language, articles from the period 2000–2021, experimental and/or control groups, non-pharmacological approach, implementation of non-pharmacological methods for elderly groups diagnosed with dementia, stimulation of visual, auditory, or motor skills, evaluation of caregivers and beneficiaries. The exclusion criteria we used were: written in a language other than English, non-experimental and/or control groups, implementation of only pharmacological methods for elderly groups diagnosed with dementia, sole assessment of careers of people with cognitive impairment.

**Figure 1:**
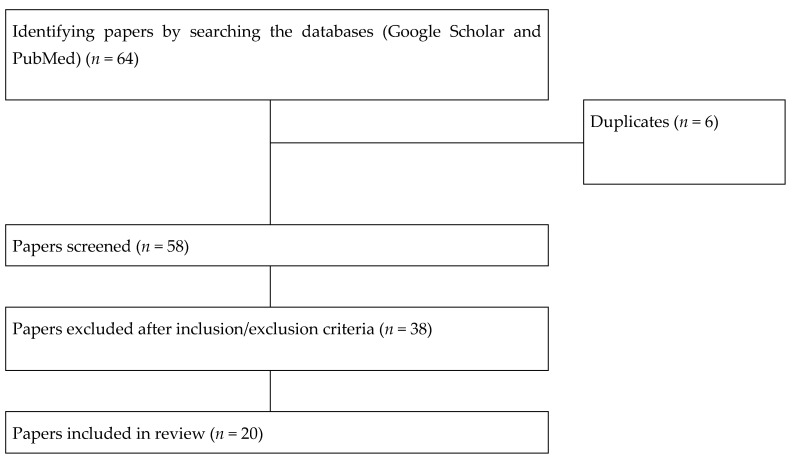
Flowchart overview of the results; how papers were retrieved, screened, and included.

Given that the results were heterogeneous, we decided that this paper would review literature in narrative style rather than a systematic one. To better understand how the articles were selected, we inserted a flow chart (Figure 1), two tables with the relevant studies for each type of therapy, Table 1 and Table 2 for musical therapy, respectively Table 3 and Table 4 for art therapy.

## 3. Results

### 3.1. Caregiver Burden

A study that administered the Caregiver Burden Inventory (CBI) to 86 caregivers of patients with AD showed a direct link between the severity of the disease and caregiver burden. The study also showed that the caregiver role was often taken by the wife or daughter of the patient with AD.

Studies have shown that there are many repercussions on the physical and mental health of caregivers of patients with AD, as well as altered family relationships, job loss followed by financial difficulties, and even an increase in mortality.

Female caregivers often become physically, emotionally, and financially overwhelmed. Their time is permanently restricted, and they can lose a number of opportunities. They generally look for strategies based on emotions, making their burden even more difficult [14].

It is well-known that a good medical and care service benefits the elderly by improving their self-esteem, quality of life, and mental health, and according to recent studies, these benefits are also reported among the family members [15].

### 3.2. Music, a Form of Therapy

Throughout history, with the desire to improve the effects of drug therapy, steps have been taken to develop a complex care plan to increase people’s quality of life. In 1997, Cohen-Mansfield and Werner stated that to improve the daily lives of the elderly in a residential center, they must be involved in activities that are enjoyable but also stimulating [16]. Different studies have tried to demonstrate the effectiveness of music in various psychiatric pathologies, including dementia. Since art is more conductive to qualitative than quantitative evaluations, studies on this subject are challenging.

In 2010, a randomized clinical study (RCT) was conducted by Cooke et al. on the influences of music therapy on people with dementia who also experienced behavioral disorders and anxiety. Their results indicated that interventions such as music therapy or reading therapy had minimal benefit, and only some of the participants showed any improvements [17]. 

However, improvements in speech, behavior, and depressive symptoms through music therapy interventions have been demonstrated by Brotons, M. and Koger, S.M. The previously mentioned study demonstrated that speech content and fluency as evaluated by the spontaneous speech subscale of the WAB (Western Aphasia Battery) were superiorly improved following musical therapy than as a result of oral sessions with a specialized therapist [18].

There is an ongoing randomized parallel-design controlled trial with the aim of assessing the effects of reminiscence therapy on cognitive, emotional, behavioral, and psychological symptoms, daily living activities in patients with dementia in addition to conventional drug treatment., musical therapy also being investigated [19].

The efficacy of individualized recreational therapy was shown to be beneficial to dementia-suffering subjects by reducing their disturbing behavior described as passive (lack of motivation or initiative), agitated (wandering, verbal or physical aggression) or mixed [20]. Minimizing the agitation of elderly patients through either calming music and hand massage or a combination of both has been explored in a nursing home setting and the results suggest that interventions performed separately offer the same improvement as combining the two types of interventions [21].

A 2004 randomized control study evaluated the state of mood and cognitive function in women with dementia before and after performing music-based physical exercise interventions and concluded that measurements of both Mini-Mental State Examination (MMSE) and the Amsterdam Dementia Screening Test 6 (ADS 6) were improved as a result of the above-mentioned therapy, these findings are supported by a similar study done in Taiwan by Sung H.C. [22,23]. Engaging nursing home residents diagnosed with Alzheimer’s disease in recreational activities such as games and songs, which encouraged hand to eye coordination, range of movement, cognitive, respiratory and circulation functions have yielded unsatisfactory results in the long term, whereas the state of effect during and immediately after the activities has only shown modest signs of improvement [24]. Short-term improvements have also been observed during a case-control study carried out by H. B. Svansdottir and J. Snaedal focused on the dynamics before and after musical therapy intervention of activity disturbances, aggressiveness and anxiety. Delusional ideation suffered no improvement. The effect had subsided 4 weeks after the therapy was halted [25].

Throughout history, aspects such as mood swings have been studied, with the predominant presence of depression among the elderly. Specialized studies have associated depression with changes in the level of cortisol in the human body. Corticosteroids are hormones that play an essential role in the human brain and have been associated with noticeable changes in areas such as mood, eating and gregarious instincts, nictemeral rhythm, and cognitive function. The main glucocorticoid, cortisol, can cross the blood-brain barrier due to its lipophilic structure and has the ability to cause changes in the hypothalamic-pituitary-adrenal axis. Multiple studies have shown a connection between elevated cortisol levels and the symptoms associated with Alzheimer’s dementia [26,27,28], particularly affective symptoms. Elevated levels of cortisol in institutionalized older adults were described by Holland et al. [29]. In 2013, data were published from an RCT by Chu et al. on the cortisol level in the saliva of elderly people included in a music therapy program, and although the data were not concrete in this respect, an improvement in disposition was described, which was not reversed after the study ended [30].

According to data published in 2018 by Lyu et al., the use of music therapy in approximately 300 patients with Alzheimer’s dementia proved effective compared to alternative techniques, such as reading music lyrics, and the quality of life of their members improved at the same time [31]. 

Psychomotor agitation is one of the problems faced by specialists who care for people with dementia of any kind, and these episodes cause genuine discomfort. Thus, several studies [12,32,33] have conducted randomized investigations in multiple elderly centers using a person-centered approach, in which the music therapist applied several series of sessions for up to 18 weeks, depending on the study. The results confirmed that the positive effect of music therapy was significant and associated with improvements in disruptive behavior and a decrease in the number of psychotropic substances used during therapy. However, data are insufficient to support the hypothesis of long-term improvement.

It is recognized that the right cerebral hemisphere controls certain artistic behaviors and abilities. Researchers have observed that the functional musical residue is maintained in people with neurocognitive disorders and aphasia due to injuries in the left hemisphere. Thus, some specialists in the medical field have decided to use music therapy not only for the beneficial effects on mood symptoms and social skills but also as an adjunct in oral rehabilitation and respiratory control [34,35]. Thus, once involved in musical activities, patients can develop certain skills and simultaneously engage in respiratory gymnastics.

Throughout history, medical research in the field of cognition has existed to facilitate potential therapeutic interventions, and several blood biomarkers have been discovered that have a recognized involvement in the development of neurocognitive disorders. In particular, the studied neurodegenerative elements include β-amyloid plaques, plasma levels, and leukocyte telomeres. According to some studies, the accelerated aging process in Alzheimer’s dementia is associated with short leukocyte telomeres, although further investigation is needed, and there is no unanimous consensus to fully support the hypothesis. Randomized studies were performed to evaluate these hypotheses, such as the research conducted by Innes et al. on the influence of alternative therapies, such as music therapy and meditation, on blood biomarker levels and improvement in quality of life and behaviors. However, further investigations are needed [36,37,38,39,40,41].

In Table 1 we inserted the relevant studies for musical therapy and in Table 2 we showed study characteristics regarding musical therapy [17,18,19,20,21,22,23,24,25,30,31,32,33,40,41].

### 3.3. Art Therapy

According to the literature, art can be a reliable companion in patients with progressive dementia. Art therapy is used as an adjunct to general therapeutic measures, which should theoretically help to slow cognitive impairment, maintain functional control, and improve quality of life in people with Alzheimer’s dementia [35].

Art is also used as a form of therapy not only to correct the disabilities encountered in dementia but also to engage patients’ abilities. The function of the visual cortex is relatively well preserved in Alzheimer’s dementia, and the occipitofrontal ventral flow of recognition is maintained; therefore, the preservation of sensory and motor functions in the cortex with delayed deficits in visual and motor functions enables the production of art, even if it is not at a high level but instead has an abstract touch. The expression of positive emotions and well-being is facilitated by the relatively good preservation of the limbic system [42].

Relevant studies for art therapy were included in Table 3 and study characteristics regarding art therapy were shown in Table 4 [43,44,45,46,47].

## 4. Discussion

The reviewed literature suggests that the use of unconventional, non-pharmacological treatments as alternative therapies might prevent or delay the altered quality of life of patients with Alzheimer’s dementia. Well-organized clinical investigations are still needed in order to support such a hypothesis. Most of the reviewed studies used relatively small samples of participants, so obtaining relevant statistics in this regard is challenging. Another impediment is the use of different protocols in pre-, intra-, and post-therapy evaluations due to the timing and lack of well-defined tools for such an analysis. Careful assessments of the stage of the disease and the level of cognitive impairment are necessary to decide on the best form of therapy, given that the evolution is progressive and that skills are lost along the way. Patients in the mild or mild/moderate stages can perform activities that are often difficult in advanced stages, in which a different type of care is required and sensory and motor dysfunctions are much more severe.

Randomized controlled trials have noted that, although the cognitive decline is continuous, the use of various forms of art therapy is associated with a measurable improvement in assessment scale scores in terms of quality of life in adults with Alzheimer’s dementia [43,44,45,46,47,48]. Both art therapy and music therapy seem to psychosomatically influence people with a form of dementia [49].

In this paper, we divided these forms of therapy into two categories: music therapy and art therapy, as the latter involves motor and sensory functions together (e.g., painting and drawing). In the category of art therapy, we observed the benefits of creative activity through the use of cognitive and motor functions, and in the category of music, there was a reduction in anxiety, depression, and to a lesser extent, behavioral symptoms such as aggression.

The use of unknown abilities through rediscovery or reminiscence has been discussed in the literature as making a considerable contribution to promoting a form of mental well-being [35,49,50].

The use of music therapy or a form of art therapy is reliable, given that they are non-invasive and can be performed at minimal cost.

Other important aspects of using such therapeutic methods include decreased isolation, group membership, socialization, and maintenance of social status.

## 5. Conclusions

Music therapy is a promising area that can be considered a safe and well-tolerated intervention for patients with Alzheimer’s dementia. Data are sufficient to support the positive effects of music therapy on emotional expression, relationships, and non-cognitive symptoms by reducing cortisol levels and, as a result, reducing symptoms of anxiety, depression, and to a lesser extent, behavioral symptoms such as aggression in elderly patients with dementia. However, methodological limitations were detected. Further investigations are needed to prove the long-term benefits.

With the use of art as a form of therapy, it has been observed that, although cognitive function progressively decreases over time, this form of therapy stimulates the person affected by dementia at the sensory level and could produce subjective benefits.

Art therapy has the advantage of improving cognitive function, including information processing, visual-spatial attention, and episodic memory, and also enhances and encourages the patients’ remaining abilities by stimulating the visual cortex. Furthermore, it can provide the person with dementia, as well as caregivers, an opportunity for self-expression, allowing them to depict their thoughts and emotions. The recipients of these therapies can also share memories through painting, drawing, and other creative projects.

There is a growing recognition that art and music can contribute to the quality of life of Alzheimer’s disease patients, and through these interventions, greater satisfaction and well-being may be possible.

Patients with dementia who engage in music and art therapy have shown improvements in their quality of life. Therefore, these therapies constitute a highly promising path that requires further study and attention from the medical field and social workers.

## Figures and Tables

**Table 1:** Relevant studies for musical therapy.

Alternative Therapy	Diseases or Neurological Disorder	Effects on Patients	Effects on Caregivers	Reference
Group music program	Mild-moderate dementia	Increased verbalization behavior No significant effect on agitation and anxiety	N/A	Cooke M.L., 2010 [17]
Music or conversation interventions	Dementia	Improvement in speech content and fluency after music sessions	N/A	Brotons M, 2000 [18]
Reminiscence therapy (including music)	Dementia Alzheimer’s disease	Evaluation of ADAS-Cog, CSDD, NPI, Barthel Index	N/A	Li, M., 2017 [19]
Home therapeutic recreation intervention	Dementia Agitation Passive behavior	Reduced disturbing behaviors	Respite period communication, emotional support	Fitzsimmons, S.; Buettner, L.L., 2002 [20]
Calming music, hand massage	Dementia	Reduced agitation	N/A	Remington, R., 2002 [21]
Music-based exercises	Moderate or severe dementia	Improvement in cognition	N/A	Van de Winckel A., 2004 [22]
Group music with movement intervention	Dementia	Decreased in agitated behaviours	N/A	Sung, H., 2006 [23]
Recreational activities (including musical therapy)	Dementia	Icreased mood and level of consciousness	N/A	Schreiner, A.S., 2005 [24]
Musical therapy	Moderate or severe Alzheimer’s disease	Reduced agitation and anxiety	N/A	Svansdottir HB, Snaedal J., 2006 [25]
Music therapy	Dementia Depression Cortisol Levels	Reduced depression Improved shot-term recall function	N/A	Chu H., 2014 [30]
Musical therapy	Dementia	Cognition, psychiatric symptoms, Daily activities	N/A	Lyu J., 2018 [31]
Music therapy	Dementia	Decreased agitation disruptiveness and psychotopic medication	N/A	Ridder H.M., 2013 [32]
Music therapy Recreational activities	Dementia	Short-term decrease in agitation	N/A	Vink A.C., 2012 [33]
Music therapy (ML) Kirtan Kriya meditation (KK)	Dementia Cognitive decline Telomere length (TL), telomerase activity (TA), and plasma amyloid-β (Aβ) levels	KK group increases Aβ40, improvement in cognitive and psychosocial status, improvements in stress, mood, QOL	N/A	Innes K.E., 2018 [40]
Music-based therapeutic interventions	Dementia	Low improvement in depressive symptoms, QOL No improvement in agitation or behavioral disorder	N/A	van der Steen J.T., 2017 [41]

N/A: not available; ADAS-Cog: The Alzheimer’s Disease Assessment Scale–Cognitive Subscale; SDD: Cornell Scale for Depression in Dementia; NPI: neuropsychiatric inventory; KK: Kirtan Kriya meditation; Aβ40: plasma amyloid-β40; QOL: Quality of life

**Table 2:** Studies characteristics regarding musical therapy.

Study First Author, Year	Design	Study Focus	Intervention Type	Duration	Outcomes Measures
Cooke M.L., 2010 [17]	Randomized cross-over controlled study	Dementia	Experimental group: music therapy interventions (listening)	40 min, three mornings a week for eight weeks	CMAI-SF, RAID
Brotons M, 2000 [18]	Randomized controlled trial	Alzheimer’s and related disorders	Music therapy vs. conversation sessions	Twice per week for 20–30 min for a total of 8 sessions	MMSE, WAB, AQ
Li, M., 2017 [19]	Randomized controlled trial	Dementia, Alzheimer’s disease	Reminiscence therapy (including musical therapy)	35 to 45 min, 2 times/week for 12 consecutive weeks	ADAS-Cog, CSDD, NPI, Barthel Index
Fitzsimmons, S., Buettner, L.L., 2002 [20]	Quasi-experimental study	Dementia and disturbing behaviors	Individualized recreational therapy interventions	Two weeks of daily, individualized recreational therapy interventions (TRIs)	MMSE, CMAI, Passivity in Dementia Scale
Remington, R., 2002 [21]	Randomized cross-over controlled study	Dementia, Agitated behavior	Experimental	10 min exposure to either calming music, hand massage, or calming music and hand massage simultaneously	CMAI, Ward Behavior Inventory, Confusion Inventory
Van de Winckel A., 2004 [22]	Randomized controlled trial	Dementia	Experimental	3 months of daily physical exercises supported by music for 30 min/session	MMSE, ADS 6, BOP Scale
Sung, H., 2006 [23]	Randomized controlled trial	Dementia	Experimental group receiving group music with movement intervention	30 min, twice a week for 4 weeks	Modified CMAI, Likert Scale
Schreiner, A.S., 2005 [24]	Randomized control trial	Dementia Alzheimer’s	Structured observation	Structured recreation activities (including musical therapy)	Philadelphia Geriatric Centre Affect Rating Scale, MMSE
Svansdottir H.B., Snaedal J., 2006 [25]	Case-control study	Moderate or severe Alzheimer’s disease	Experimental group: music therapy interventions	18 sessions of music therapy, each lasting 30 min, three times a week for 6 weeks	BEHAVE-AD
Chu H., 2014 [30]	Randomized controlled trial	Dementia, Depression	Experimental group: music therapy interventions (listening, singing, playing instruments)	30 min sessions/twice a week, 6 weeks	CSDD
Lyu J, 2018 [31]	Randomized controlled trial	Dementia	Experimental group: music therapy interventions (listening, reading, singing)	30–40 min, twice a day for three months	MMSE, WHO-UCLA AVLT verbal fluency test, NPI, and Barthel Index
Ridder H.M., 2013 [32]	Randomized control study	Dementia, Agitation	Experimental group: music therapy interventions (listening, singing, dancing)	On average, 12 sessions of 33.8 min	CMAI, ADRQL, MMSE
Vink A.C., 2012 [33]	Randomized controlled trial	Dementia	Experimental group: music therapy interventions (listening, singing, dancing, playing an instrument)	34 sessions, 40 min on average for 4 months	CMAI, GDS
Innes K.E., 2018 [40]	Randomized controlled trial	Dementia	Kirtan Kriya meditation vs. music listening program	12-week, 12 min/day	Telomere length (TL), telomerase activity (TA), and plasma amyloid-β (Aβ) levels, QOL
van der Steen J.T., 2017 [41]	Randomized controlled trial	Dementia	Music-based therapeutic interventions	N/A	Emotional well-being and quality of life

CMAI-SF: The Cohen–Mansfield Agitation Inventory-Short Form; RAID: The Rating Anxiety in Dementia; MMSE: The Mini–Mental State Examination; WAB: The Western Aphasia Battery; AQ: The Alzheimer’s Questionnaire; ADAS-Cog: The Alzheimer’s Disease Assessment Scale-Cognitive Subscale; CSDD: Cornell Scale for Depression in Dementia; NPI: Neuropsychiatric Inventory; CMAI: The Cohen–Mansfield Agitation Inventory, ADS-6: Amsterdam Dementia Screening Test 6; BEHAVE-AD: Behavioral Pathology in Alzheimer’s Disease Rating Scale; WHO-UCLA AVLT: World Health Organization-University of California-Los Angeles Auditory Verbal Learning Test; ADRQL: The Alzheimer’s Disease-Related Quality of Life; GDS: The Geriatric Depression Scale.

**Table 3:** Relevant studies for art therapy.

Alternative Therapy	Diseases or Neurological Disorder	Effects on Patients	Effects on Caregivers	Reference
Art therapy vs. calculus	Mild Alzheimer’s disease	Improved QOL and vitality	N/A	Hattori H., 2011 [43]
Visual art training	Dementia	No quantitative benefits on overall cognition, working memory, or delayed recall	N/A	Johnson K.G., 2020 [44]
Art activity	Early-stage Alzheimer’s disease or related cognitive disorders (ADRD)	Cognitive stimulation, social connections, improved self-esteem	Social and cultural experience	Flatt, J.D., 2015 [45]
Art therapy	Dementia	Improvement in episodic memory and fluency, improved mood, confidence, and reduced isolation	Shared experience, support	Eekelaar, C., 2012 [46]
Art-based interventions	Dementia	Improvement in general cognition and functioning	Mutual support network, active involvement in group activity	Savazzi F., 2020 [47]

N/A: not available; QOL: quality of life; ADRD: Alzheimer’s disease-related dementias.

**Table 4:** Studies characteristics regarding art therapy.

Study First Author, Year	Design	Study Focus	Intervention Type	Duration	Outcomes Measures
Hattori H., 2011 [43]	Randomized controlled trial	Dementia, Alzheimer’s disease	Art therapy and control (learning therapy using calculation) groups	Once weekly for 12 weeks	MMSE, QOL, Apathy Scale
Johnson K.G., 2020 [44]	Randomized controlled trial	Dementia	Visual art training	1 h/day, 2 days/week, 8 weeks, 16 sessions	MoCA, Backward digit span task
Flatt, J.D., 2015 [45]	Randomized controlled trial	Early-stage Alzheimer’s disease or related cognitive disorders	Experimental group: art museum engagement activity	Four art engagement activity sessions	Satisfaction survey
Eekelaar, C., 2012 [46]	Exploratory study	Dementia	Art therapy	Viewing of paintings in a public art gallery, followed by an art-making visual response	MMSE, Semi-structured Interview
Savazzi F., 2020 [47]	Quasi-experimental design study	Dementia, Alzheimer’s disease	Art-based intervention	14 sessions	ADAS-Cog, QOL, NPI

MMSE: The Mini-Mental State Examination; QOL: Quality of life; MoCA: Montreal Cognitive Assessment; ADAS-Cog: The Alzheimer’s Disease Assessment Scale-Cognitive Subscale; NPI: Neuropsychiatric Inventory.

## Data Availability

The data presented in this review are available within the article.
